# A Multimedia Mobile Phone–Based Youth Smoking Cessation Intervention: Findings From Content Development and Piloting Studies

**DOI:** 10.2196/jmir.1007

**Published:** 2008-11-25

**Authors:** Robyn Whittaker, Ralph Maddison, Hayden McRobbie, Chris Bullen, Simon Denny, Enid Dorey, Mary Ellis-Pegler, Jaco van Rooyen, Anthony Rodgers

**Affiliations:** ^2^Department of Community PediatricsUniversity of AucklandAucklandNew Zealand; ^1^Clinical Trials Research UnitUniversity of AucklandAucklandNew Zealand

**Keywords:** Smoking cessation, cellular phone, learning

## Abstract

**Background:**

While most young people who smoke want to quit, few access cessation support services. Mobile phone–based cessation programs are ideal for young people: mobile phones are the most common means of peer communication, and messages can be delivered in an anonymous manner, anywhere, anytime. Following the success of our text messaging smoking cessation program, we developed an innovative multimedia mobile phone smoking cessation intervention.

**Objective:**

The aim of the study was to develop and pilot test a youth-oriented multimedia smoking cessation intervention delivered solely by mobile phone.

**Methods:**

Development included creating content and building the technology platform. Content development was overseen by an expert group who advised on youth development principles, observational learning (from social cognitive theory), effective smoking cessation interventions, and social marketing. Young people participated in three content development phases (consultation via focus groups and an online survey, content pre-testing, and selection of role models). Video and text messages were then developed, incorporating the findings from this research. Information technology systems were established to support the delivery of the multimedia messages by mobile phone. A pilot study using an abbreviated 4-week program of video and text content tested the reliability of the systems and the acceptability of the intervention.

**Results:**

Approximately 180 young people participated in the consultation phase. There was a high priority placed on music for relaxation (75%) and an interest in interacting with others in the program (40% would read messages, 36% would read a blog). Findings from the pre-testing phase (n = 41) included the importance of selecting “real” and “honest” role models with believable stories, and an interest in animations (37%). Of the 15 participants who took part in the pilot study, 13 (87%) were available for follow-up interviews at 4 weeks: 12 participants liked the program or liked it most of the time and found the role model to be believable; 7 liked the role model video messages (5 were unsure); 8 used the extra assistance for cravings; and 9 were happy with two messages per day. Nine participants (60%) stopped smoking during the program. Some technical challenges were encountered during the pilot study.

**Conclusions:**

A multimedia mobile phone smoking cessation program is technically feasible, and the content developed is appropriate for this medium and is acceptable to our target population. These results have informed the design of a 6-month intervention currently being evaluated for its effectiveness in increasing smoking cessation rates in young people.

## Introduction

Many young people want to stop smoking [1-3], but few access traditional cessation services [4]. Currently available services generally do not cater to young smokers, who tend to value confidentiality and anonymity, ease of access, proven efficacy, and the use of peers in such services [5,6]. Due to the ubiquity of mobile phone use by young people, we developed a Short Message Service (SMS), or text message, smoking cessation intervention that was tested in the STOMP (STOp smoking by Mobile Phone) study in 2001 [7,8]. Short-term quit rates were doubled among the intervention group compared to the control group. This intervention is soon to be implemented in several countries, including New Zealand, where it is being provided as a government-funded and universally available program.

Since STOMP, the introduction of multimedia mobile phones has made it technically possible to expand the content of such programs. Video message technology provides an ideal opportunity for the use of role modeling, or observational learning, which involves watching others perform a task or behavior. According to social cognitive theory, individuals gain socialization information and cognitive skills from observational learning and are likely to remember and repeat the behaviors provided by a model [9,10]. There is growing evidence from nonexperimental clinical studies of the effective use of role modeling in behavioral change [11], sports medicine and injury rehabilitation [12,13], and a variety of clinical contexts [14-23]. Role modeling by parents and peers is thought to be a key factor in smoking initiation by young people [24-27].

Video-based smoking cessation “education” programs have been trialed with modest increases in quit rates [28-30]. The benefits specified by participants included seeing others quit smoking, dealing with stress and bad feelings, talking about what to do with urges to smoke, and observing ways to get peer support [31,32].

In particular, “coping role models” may be useful in smoking cessation. The observer watches a person going through a quit attempt who presents various coping strategies for dealing with the difficulties in changing behavior [33]. The observer picks up relevant cues and information that may increase self-efficacy, motivation, and problem-solving skills and therefore increase the likelihood of his or her quit attempt being successful. Model similarity, in which the observer identifies with the role model with respect to age, gender, culture, and language [34,35], is likely to be important as reflected in youth development principles [36].

Furthermore, mobile phone–based programs can easily incorporate known effective smoking cessation techniques, such as individuals setting their own quit date, goal setting, reminders and motivational messages, advice, and information on what has been shown to be effective. These messages can be sent automatically and can be received anytime, anywhere and completely independent of location.

We hypothesized that a multimedia mobile phone smoking cessation program would increase abstinence rates in young smokers who want to quit compared to a control intervention. The use of new mobile technology in itself may appeal to young people and thereby encourage participation; however, the program content must be varied, relevant, and appropriate in order to maintain interest. Therefore, input from young people into the development of the content for the program was essential. This paper describes the steps in development of the program content for this multimedia mobile phone–based smoking cessation intervention. It includes the results of a pilot study designed to test the system and obtain feedback from participants.

## Methods

**Figure 1:**
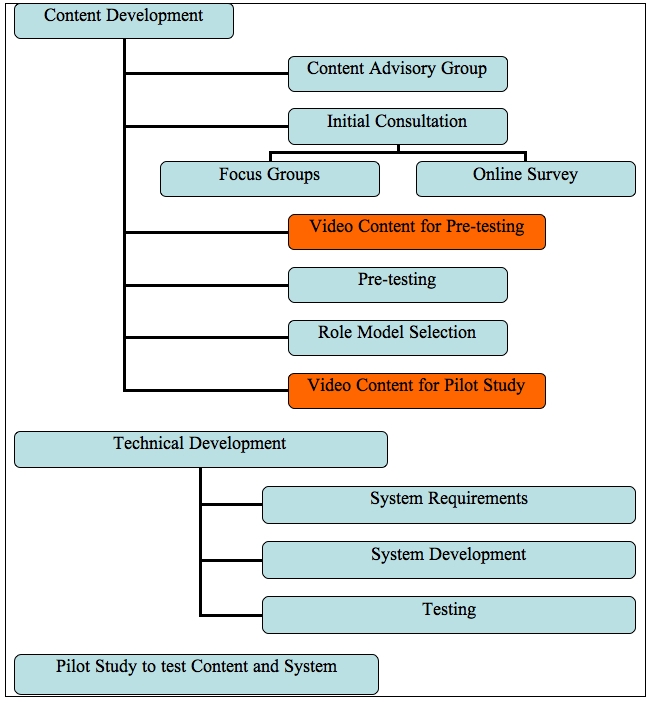
Steps in program development

Figure 1 outlines the steps in the development of the program. Content development and technical development are discussed in further detail below..

### Content Development

#### Content Advisory Group

We convened a content advisory group with members selected to provide expert advice on smoking cessation, youth health, Maori (the indigenous population of New Zealand) health, public health, psychology, social marketing, media, TV production, and mobile phones. This group met monthly and began by developing the themes of role modeling, youth development principles, and effective smoking cessation interventions. The group also reviewed the results of all assessments described below, gave input into scripts, and reviewed all videos produced.

#### Initial Consultation

Initial consultation included four focus groups of students (16-18 years, smokers and nonsmokers) in a metropolitan, multicultural college in order to obtain a breadth of information on current and potential uses of mobile phones among young people. Participants were randomly selected from the school roll and were invited to participate by school staff. Questions were pre-set (see Textbox 1), and the format was standard across groups. An independent survey research unit at the University of Auckland facilitated the focus groups, audiotaped and transcribed the discussions, and undertook a general thematic analysis.

**Examples of focus group questions**

What do you use your mobile phone/s for? What sorts of things do you like to do with your mobile phone?

Prompts: Listen to music, play games, surf the Net, download ringtones, video calling, watch videos/TV, look at cartoons, text, call

What sorts of things do you like to do to relax? What would you most like to receive over your mobile phone to help you relax?

Who would you most like to watch working through similar problems to see how they cope?

Prompts: People like you (same age/circumstances)? Famous people? Someone you know? Someone older than you who has been there?

What do you do when you feel you need/want support?

Prompts: Who do you go to? (Who is most likely to provide support?)

How do they support you? What do they do to support you?

Could mobile phones be useful in getting social support? How?

The final program will target all young adults aged 16 years and over (with no upper age limit); therefore, we also consulted a slightly older age group via an online survey. Participants were recruited from the website of a popular Auckland radio station (Mai FM) oriented to young adults. The survey was open to all those aged 16 years and over who owned a mobile phone. Questions were asked about their current mobile phone use and interest in potential uses of mobile phones for health programs. A quantitative analysis of these results was undertaken.

#### Pre-Testing

In order to obtain a range of video material to be pre-tested, media students at tertiary media training institutions were invited to submit their own video and animated content. These students were given a 1-page brief on the smoking cessation program to be developed. However, only a small number of videos were submitted. Further videos were sought by advertising with Student Job Search, a popular service among young people seeking part-time employment, with a very brief description of what was required (a 30-second video clip to help young people who want to quit smoking).

From these, four video clips were selected by the Content Advisory Group to best represent the different styles of video that had been submitted. Young people recruited via the radio station’s website were invited to complete an online survey to compare these videos (embedded in the online survey) with a professionally made video of a young quitter. Participants rated each video out of 10 according to how much they liked it, as well as answering more specific questions about each video and placing them in order of preference.

#### Role Model Selection

Next, potential role models (smokers or ex-smokers only) for the pilot study videos were recruited from Student Job Search. Twenty-seven audition videos in which each role model talked about smoking or quitting were shown to young people in a variety of settings (university students between classes, workers at a predominantly young adult workplace, and those who had previously expressed an interest in consulting on the program). These participants rated the role models (1-27) for credibility and whether they would want to continue watching them. They also recorded their initial impressions of each role model on paper.

The top-rated model was selected for the pilot study, and her own recent quitting experiences were converted into a chronological series of messages from a lead-up to 4 weeks after quitting. Each message was based on a particular issue associated with quitting and how the model coped with that issue, or on how to keep motivated and stay quit. These messages were recorded as approximately 30-second vignettes in a video diary style using the model’s own words. They were designed to be sent to participants twice daily, starting several days prior to their quit attempt.

We also talked to two high school drama classes about the tobacco industry and the effects of smoking, in a manner consistent with the “Truth” anti-tobacco industry media campaign [37]. The students were then assisted in producing short anti-tobacco video clips, predominantly of students talking about proven ill effects of smoking and behaviors of the tobacco industry. These videos were used to add variety to the program but were not pre-tested due to time constraints.

### Technical Development

A system to deliver the program was designed in a system requirements document by the principal investigator (RW) and staff and was translated into system development by the information technology (IT) senior developer (JvR). The video clips were then arranged into a pre-set schedule to deliver two messages per day (predominantly the role model videos with some anti-tobacco clips and a small number of text messages interspersed) in a chronological sequence starting with a lead-up to Quit Day, messages for Quit Day, and then post–Quit Day.

The program began with online registration and an automated text message to those eligible to participate. An appropriate reply to this message provided evidence of informed consent to participate in the study. This system then guided the participant to select a Quit Day and two appropriate time bands (per 24-hour clock) to receive messages from the program. A schedule for program delivery to that participant would then be set by the system.

The video messages were hosted on a wireless application protocol (WAP) site. At a random time within the selected time band, a text message with the uniform resource locator (URL) for the appropriate video message was sent to each participant. By scrolling over or highlighting the URL within the text message, the video message would begin automatic download and then play on the phone. Video messages could be viewed immediately upon receipt or at a later time as appropriate.

As well as the pre-set program of messages, participants could request extra support messages on demand by texting a keyword to the program shortcode (a 4-digit number). Tips on managing cravings would then be automatically and immediately sent to the participant. This function (CRAVE) had been popular with some participants in the previous text message program [7].

### Pilot Study

A pilot study was conducted from June to August 2007 to test the developed program and the delivery system and to obtain feedback from participants regarding their satisfaction with the program. Participants for the pilot phase were recruited by radio commercials (Mai FM) or via a direct link from the radio station’s website. To be eligible, participants were required to have a video message–capable mobile phone on the Vodafone network (which has approximately half of the mobile phone market in New Zealand), be 16 years of age or older, be a daily smoker who wanted to quit, and be a resident in the Auckland region (population 1.2 million).

Potential participants registered online by self-completing an eligibility questionnaire and were provided with a participant information sheet and consent form (or could request to have them mailed or emailed). Eligible participants then received an automated consent text message and were required to reply “I consent.” Upon receipt of the appropriate response, the system directed participants by text message to return to the website and complete registration details. All participants received two messages per day—the role model quit diary interspersed with the anti-tobacco videos and text messages (see the Multimedia Appendix for a sample role model video clip). At the end of the 4-week program, participants were called by study staff to complete a telephone questionnaire. There was no cost to participants to take part in the study.

All study procedures and documents were approved by the Ministry of Health’s Ethics Committee.

## Results

### Content Development

#### Initial Consultation

Four focus groups comprised 27 college students aged 16-18 years. Groups were stratified by gender and ethnicity (Maori/Pacific and Indian/other; in New Zealand, “Indian” represents a mix of people from India and Fijian-Indians). Findings from the focus group discussions demonstrated that all of the participants used mobile phones regularly, and all groups expressed an interest in the idea of a mobile phone program to support them in dealing with any particular issues they may face. Text messaging was considered to be potentially useful for positive reinforcement messages and providing information. Listening to music or music videos was the preferred mobile phone feature for helping them to relax. Although less frequently mentioned, jokes, funny pictures, and games were also perceived to be potential tools to aid relaxation and provide distraction. Some female students also wanted to watch movies and soap operas. A consistent finding across the groups was that videos and cartoons would be useful to illustrate strategies for dealing with problems. Participants, however, clearly expressed that it would be important that the characters shared similar ethnic characteristics and be a similar age. Video calling was not perceived to be a useful component of an intervention, with some students clearly articulating a dislike for the loss of anonymity and the potential of being identified.

Of 172 online surveys submitted, 19 were excluded (four incomplete, 13 underage, two duplicates). The average age of participants was 24 years (range 16-52). Nearly half (48%, n = 74/153) were of European ethnicity, 25% (n = 38) were Maori (indigenous New Zealanders), and 15% (n = 23) were Pacific Islanders. Just over a third of participants understood that their phones were capable of viewing video messages, and of these, over half were actually sending and receiving video messages at least weekly. Table 1 summarizes the participants’ preferences for program content that were expressed in the online survey.

**Table 1:** Online survey results of participants’ preferences for program content

	% (No.)
**What sort of things do you like to do on your mobile phone? (could select more than one)**	
Text message (SMS)	99 (151)
Call	90 (137)
Play games	59 (91)
Enter competitions	41 (62)
Download from the Internet	40 (61)
Listen to music	38 (58)
Surf the Net	27 (41)
Watch videos	25 (38)
Look at jokes	18 (28)
Video calls	9 (14)
Watch cartoons	3 (5)
**What would you most like to receive over your mobile phone to help you to relax? (could select more than one)**	
Music	75 (114)
Videos	35 (53)
Games	30 (46)
Jokes	26 (40)
TV	26 (40)
Competitions	30 (46)
Cartoons	16 (24)
Nothing	12 (19)
**If you were to sign up to receive video messages over your phone to help you be healthier (eg, stop smoking, exercise more), how many such messages would you want to get each day?**	
One message per day	42 (64)
Less than one message per day	28 (43)
Two to five per day	20 (31)
More than five per day	9 (14)
**If you were part of such a program, how would you like to interact with others going through the same program with you? (could select more than one)**	
Writing a blog	27 (41)
Reading someone’s blog	36 (55)
Writing messages on a message board	35 (53)
Reading messages	40 (61)
Being paired with a “buddy”	29 (45)
Prefer no contact	18 (27)

#### Pre-Testing

A total of 41 participants, with an average age of 24 years (range 16-45), completed the pre-testing online survey with embedded video clips (another three started but did not complete the survey): 29% were smokers, 22% were Maori, and 76% were female. Of these participants, 37% stated that they would prefer to see animated clips, 27% casual interviews, 24% a mix of music/images/videos, and 10% studio interviews. Of all the clips in the survey (including one animation), participants ranked the casual interview clip the highest. With respect to the videos, the person in the video clip was more important than the style of the clip. In particular, how “real,” credible, and honest the person was perceived to be and whether the participant related to him or her personally were key factors. The technical quality of the clips (particularly the sound) was also important, and the duration of the included videos (30-45 seconds) was seen as appropriate.

### Technical Development

A significant technical challenge occurred during the pilot study when the mobile communications network inadvertently charged participants to download the video clips, meaning that those participants using prepaid cards (or “pay-as-you-go”) with no credit on their phones at the time were unable to view the clips. This issue was soon rectified by the network, and all participants were reimbursed. The online registration forms also occasionally failed to open at the beginning of the pilot, and this may have resulted in two potential participants failing to complete registration. The technical challenges experienced by the participants in the pilot study are summarized in Table 2.

**Table 2:** Technical challenges (N = 13)

	No.
**Were there any technical issues that you experienced?**	
No	4
Yes	9
**If yes, what were the issues?**	
Couldn’t open the link due to the credit issue	2
Mobile network coverage not always available	3
Couldn’t open the link, didn’t know how	4
**What did you do about this? (if couldn’t open the link)**	
Emailed for advice	1
Worked it out myself	2
Gave up (until phoned by study staff)	1

### Pilot Study

For the pilot study, 17 participants completed the full registration over a 5-week period; however, two participants withdrew before viewing the program (one due to the credit issue described above and one was unable to be contacted to find out the reason). Of the 15 who received the program, 13 were followed up after 4 weeks (two were unable to be contacted despite multiple attempts). Table 3 summarizes the characteristics of the pilot study participants.

**Table 3:** Pilot study participant characteristics (N = 17)

Characteristic		No. (%)
**Age (years)**		
	16-19	2 (12)
	20-24	9 (53)
	25-29	2 (12)
	30-34	2 (12)
	35+	2 (12)
**Sex**		
	Male	6 (35)
	Female	11 (65)
**Ethnicity**		
	Maori	6 (35)
	New Zealand European	3 (18)
	Pacific Islander	4 (24)
	Indian	2 (12)
	Other	2 (12)
**Income (NZ $)**		
	Less than 15,000	5 (29)
	15,000-30,000	2 (12)
	30,001-45,000	7 (41)
	45,001-60,000	1 (6)
	Refused to answer	2 (12)
**Smoking behavior**		
	1-10 cigarettes/day	15 (88)
	11-30 cigarettes/day	2 (12)
**Time to first cigarette**		
	0-30 minutes	5 (29)
	Over 30 minutes	12 (71)
**Previous quit attempts**		
	0	3 (18)
	1	5 (29)
	2	5 (29)
	3-5	3 (18)
	5+	1 (6)

Only one participant did not like the program. Of the 12 (92%) who stated that they liked the program or liked it most of the time, the features they liked the most were the support provided, reminders, information, encouragement, the fact that they knew messages were coming, advice, and the interesting messages and their relevance to them personally. The participants’ responses to the video diary are shown in Table 4. The majority of participants said that they could relate to what the role model was saying (n = 12) and that they found the role model to be believable (n = 12).    

**Table 4:** Participants’ responses to aspects of the pilot program (N = 13)

	No.
**Role model video diary messages**	
Liked them (Summary of comments: relevant, honest, upfront, made it more real, felt like I was not just on my own but she was going through it too)	7
Didn’t like them (Summary of comments: got bored, not professional / very basic, only good thing was a reminder, didn’t know what happened to her— if she went back to smoking, really liked the CRAVE function, would like animation/music—would be more professional)	1
Unsure (Summary of comments: she was not the same as me, good that they were not too flashy or fake but were simple, liked them at start but got sick of moaning ones, just like a story, clips could have been longer—felt “cut-off,” not sure if helpful, liked some of them, sounded similar/repetitive, got sick of them)	5
**Did you like the other (anti-tobacco) video clips?**	
Liked them	7
Liked them most of the time	1
Didn’t like them	3
**What did you think of the daily quantity of messages?**	
About right	9
Too many	1
Not enough (suggesting three or four per day)	3
**Generally speaking, when did you view the clips?**	
As soon as they arrived	5
A few minutes after they arrived	2
A few hours after they arrived	3
A mixture of as soon as arrived / few hours later	3

Some participants (n = 8) said that they saved the clips they liked in order to watch them again later. None forwarded clips to other people. Five participants stated that they appreciated being able to select their own times to receive the messages; however, others were happy to receive the messages anytime but watch them later when it was more convenient. Comments about the timing of messages included the following: it was not appropriate to receive messages at work; early morning and evening were good times; morning and afternoon, when cravings are strongest, were best; it was best after dinner; best during break times at work.

The ability to request messages on demand (CRAVE) as needed to deal with cravings was popular with the eight participants who used it—the remainder didn’t try it or forgot that it was available. Text messages were popular, particularly those that imparted information on smoking and quitting tips. Eight participants said that they would like more of these, and five said the amount was about right. When asked about additional components that could be included, jokes and polls/quizzes were the most popular, followed by music, then animations.

Nine participants stopped smoking during the program (Table 5), and of those who did not quit, half had cut down. All of those who quit said that they felt the program had helped them: “If I hadn’t started the program, I would still be smoking.”

**Table 5:** Smoking outcomes (N = 13)

	No.
**Did you stop smoking during program?**	
Yes	9
No	4
**If yes, do you think the program helped you to stop smoking?**	
Yes	9
**What about the program helped you the most?^a^**	
Setting a quit date, lead-up, role model clips	
Encouraging stuff, tips	
Text messages	
Something new	
Quitting tips	
Providing motivation	
Acted as a reminder to what you are doing, good to know others are also quitting at same time	
Regular updates, inspirational, mates tried to quit without it and failed, so felt good	

^a^Summary of comments from participants

## Discussion

This study breaks new ground in the eHealth arena and adds new information about young people’s interest in and perspectives on the use of new mobile phone technology as a platform for delivering health interventions. The degree of interest and support was sufficiently high for us to proceed with development of a full 6-month randomized controlled trial with greater breadth and depth of content based on the detailed feedback provided by participants so far, including the use of multiple role models and the ability to personalize the program. This trial will test the program’s effectiveness in increasing smoking cessation rates in young people compared with a control program.

The pilot study identified several technical challenges. First, clear and accessible information on how to open video clips on different mobile phone handsets needs to be provided early in the sign-up process. Second, systems that rely on a third party, in this case a telecommunications company, need to be thoroughly tested to provide assurance that these will work. Participants being charged for downloading the video clips was a significant barrier to adopting the intervention in this pilot.

Strengths of our study include the use of established theory on which to construct our program elements and format, the extensive input from young people at various steps in development, the use of key youth media to recruit participants successfully from all major ethnic groups in New Zealand, and the extent to which we were able to test content and functionality in circumstances that approximated the current production version.

### Limitations

Three main limitations were identified. First, technical issues with credit on prepaid phones and difficulties with the initial registration process restricted our ability to gain feedback from some potential participants. Second, this was a pilot study and numbers were small, so results may not be fully representative of the wider youth smoking population. Nevertheless, recruitment and registration processes were the same as those being used in the full trial, so there is no reason to believe that the pilot study participants would systematically differ from those in the main study. Third, the pilot was not designed to examine the effectiveness of the program with respect to smoking cessation outcomes, so no conclusions about these outcomes should be drawn from the results.

### Comparison With Prior Work

This research confirms our previous experiences with the STOMP text message mobile phone smoking cessation study [7,8]; that is, mobile phones are a key medium for reaching young people with health support. Second, mobile phone–based interventions allow individualization and choice, anonymity, ease of use, timely support regardless of location, and support on demand.

Others have used mobile phones for smoking cessation [38], and, more specifically, text messaging for smoking cessation in conjunction with other modalities (eg, text messaging in conjunction with a website for college students [39] and text messaging in conjunction with information packs, email, and phone calls [40]). Text messaging has also been used in other health services for appointment reminders [41] or home monitoring (eg, of blood glucose in diabetes management [42,43]). However, to our knowledge, this is the first published account of the use of multimedia technology in this way.

### Conclusions

A multimedia mobile phone smoking cessation program is feasible and acceptable to young people. We encountered relatively few technical issues, the most common being lack of knowledge on how to open the video clips, a problem easily overcome by providing more instruction. Smoking cessation content was appropriate for incorporation in video messages delivered by mobile phone and was acceptable and helpful to participants. Role models were a key factor in the appeal of the program, but they must be perceived as real, honest, and credible. With some participants becoming tired of the same model, the ability to choose between multiple role models and to vary and personalize content by selecting or de-selecting components will be important features in future iterations of this program.

## Multimedia Appendix

Example of a role model video clip

## Abbreviations

STOMP: STOp smoking by Mobile Phone
